# The Expression of CD90/Thy-1 in Hepatocellular Carcinoma: An *In Vivo* and *In Vitro* Study

**DOI:** 10.1371/journal.pone.0076830

**Published:** 2013-10-08

**Authors:** Caecilia Hapsari Ceriapuri Sukowati, Beatrice Anfuso, Giuliano Torre, Paola Francalanci, Lory Saveria Crocè, Claudio Tiribelli

**Affiliations:** 1 Centro Studi Fegato, Fondazione Italiana Fegato, Trieste, Italy; 2 Hepatology Unit, Gastroenterology and Nutrition, Department of Surgery and Transplantation, Ospedale Pediatrico Bambino Gesù, Rome, Italy; 3 Department of Laboratories, Ospedale Pediatrico Bambino Gesù, Rome, Italy; 4 Department of Medical Sciences, University of Trieste, Trieste, Italy; University of Udine, Italy

## Abstract

Although the CD90 (Thy-1) was proposed as biomarker of several tumors and cancer stem cells, the involvement of this molecule in the progression of hepatocellular carcinoma (HCC) and other less frequent hepatic neoplasms is still undefined. The distribution of CD90 was investigated both in *in vivo* (human tissues samples) and *in vitro* (human HCC cell line JHH-6). A total of 67 liver tumors were analyzed: 51 HCC, 6 cholangiocarcinoma and 10 hepatoblastoma. In all cases, paired tissue sample of both the tumor and cirrhotic liver was available. Hepatic tissue obtained in 12 healthy livers was used as control. CD90 gene expression was studied by RT-qPCR, protein expression was assessed by quantitative Western Blot, immunofluorescence and flow cytometry. The CD90 expression analysis showed a significant increment in tumor compared to both its paired cirrhotic tissue and normal liver (p<0.05 and p<0.001, respectively). This increase was accompanied by the up-regulation of stromal component in the cancer, as demonstrated by alpha smooth muscle actin staining. *In vitro* analysis of JHH-6 cell line showed a higher proliferation capacity of CD90^+^ compared to CD90^-^ cells (p<0.001), also noticed in 3D clonogenic assay (p<0.05), associated by a significant higher expression of the promoting factors (hepatocyte growth factor, fibroblast associated protein and alpha smooth muscle actin 2). A higher expression of the breast cancer resistance protein was found in CD90^+^ subpopulation while the multidrug resistance protein 1 showed an opposite behavior. Collectively, these results point to the importance of CD90 in the HCC.

## Introduction

Primary liver cancer (PLC) is the fifth most common neoplasms in the world and the third most common cause of cancer-related death. PLC accounts for around 1% of all death worldwide [1]. Approximately more than 500,000 new cases are diagnosed per year, with an age-adjusted worldwide incidence of 5.5–14.9 per 100,000 populations [2]. Hepatocellular carcinoma (HCC) accounts for around 85-95% of all PLC cases. The development of HCC is usually derived as a final consequence of long term liver injury, in which liver cirrhosis takes part as the strongest risk factor [3,4]. Most of HCC patients with intermediate-advanced stages receive systemic chemotherapies, such as conventional chemoembolization with doxorubicin [5].

The CD90 (Thy-1) had been proposed as one of the important molecules in cancer, including in HCC. CD90 is a 25-37 kDa glycophosphatidylinositol (GPI)-anchored protein expressed in many cells such as T-cells, thymocytes, neurons, endothelial cells and fibroblast. CD90 operates as an important regulator of cell to cell and cell to matrix interaction, apoptosis, adhesion, migration, cancer and fibrosis [6]. CD90 is also expressed in bone-marrow derived stem cells [7], hepatic stem/progenitor cells both in adult or fetal livers, but not in adult hepatocytes [8–10]. Cells with phenotype CD90^+^CD44^+^CD29^+^CD73^+^ isolated from normal adult liver show osteogenic and endothelial potential differentiation, and could be also induced to pancreatic islet-like structures [8]. In prostate cancer, an increased expression of CD90 was associated with the presence of cancer associated fibroblasts (CAFs) in the tumor microenvironment and served as cancer biomarker [11,12].

In liver, CD90 expression was found preferably in poorly differentiated HCC and suggested to be associated with a poor prognosis [13–15]. In relation with cancer stem cells (CSC), CD90^+^ cells but not CD90^-^ cells, obtained from HCC cell lines, tumor tissues, and peripheral blood displayed tumorigenic and metastatic capacity when injected into immunodeficient mice [16–18]. Recently, it had bene reported that based a gene ontology analysis the over-expressed genes in CD90^+^ cells from HCC were associated with inflammation, drug resistance and lipid metabolism compared to CD90^+^ from nontumoral liver [19].

In spite of the complexity of the function of CD90 in liver neoplasm, the role of this marker in the natural history of HCC is still poorly defined and needs to be expanded. In addition, this information is not available in other types of PLC. The aim of this study was to study the significance of CD90 expression in a large number of HCC and to compare with that of liver cirrhosis. *In vivo* data were compared to those obtained *in vitro* using a cell line originated from poor differentiated human HCC. The results from the two models point the importance of this molecule in the progression of HCC.

## Materials and Methods

### Samples

#### Human liver tissues

A total of 79 human liver tissues were analyzed: HCC (n=51), cholangiocarcinoma (CC, n=6), and hepatoblastoma (HB, n=10). Samples were obtained from patients undergoing liver resections or liver transplantations. Most of those included the paired tumoral and liver cirrhosis (LC) regions. Normal liver tissues from donors were used as controls (CTRL, n=12). HCC samples were obtained from 5 female and 20 male patients, 2 were HBV and 6 HCV positive, serum alpha fetoprotein (AFP) ranged from 5 to 5000 ng/ml, score of Child-Turcotte-Pugh (CTP) from A5 to C, score of Model for End-Stage Liver Disease (MELD) from 7 to 12. The histological analysis by hematoxylin-eosin (HE) staining and the final diagnosis of patients were established based on international criteria together with its clinical findings. Based on Edmonson-Steiner histological grade, 14 HCC were grade 1-2, 8 HCC were grade 3-4, and 3 HCC were undefined.

#### HCC cell line and animal

An undifferentiated human HCC cell line JHH-6 (JCRB1030) was obtained from the Japan Health Science Research Resources Bank (HSRRB, Tokyo, Japan) and used as *in vitro* model. These cells have epithelial-like structure with undifferentiated morphology and are Hepatitis B Virus (HBV) DNA negative. Cells were maintained in Williams’ *E medium* supplemented with 10% (v/v) FBS, 1% L-glutamine and 1% antibiotics at 37°C in a humidified 5% CO_2_ incubator, and were routinely sub-cultured with 0.05% trypsin in phosphate buffer at 85%-95% confluence.

Eight weeks male nude homozygote and NOD/SCID mouse were bought from Harlan Laboratories (Udine, Italy). They were maintained in animal house facility University of Trieste and received human care according to the criteria outlined in the guide for the care and use of laboratory animals.

#### Ethics

Informed consent was obtained from patient or by a legal representative and patient anonymity had been preserved. The investigation was conducted according to the principles expressed in the Declaration of Helsinki. The protocol and animal study were approved by the ethical committee of the University of Trieste and responsible administration of the Ministry of Health (Permit number: 107/2010). This study was carried out in strict accordance with the recommendations in the Guide for the Care and Use of Laboratory Animals and all efforts were made to minimize suffering.

### Total RNA isolation and Reverse Transcription

Total RNA from cell line and tissues samples was extracted using the TriReagent (Sigma Aldrich, St Louis, MO, USA) according to the manufacture’s protocol. Total RNA from tissues was obtained from snap-frozen tissues stored in -80°C. RNA was quantified at 260 nm in a DU®730 spectrophotometer (Beckman Coulter, Fullertone, CA, USA). The RNA purity was evaluated according the MIQE guidelines [20] by measuring the ratio A260/A280 with appropriate purity values between 1.8 and 2.0. The integrity of RNA was assessed on standard 1% agarose/formaldehyde gel. The reverse transcription of 1 µg of total RNA was performed with an iScript cDNA synthesis Kit (Bio-Rad Laboratories, Hercules, CA, USA) according to the manufacture’s suggestions.

### Reverse Transcription quantitative real time PCR (RT-qPCR)

The RT-qPCR was performed according to the iQ SYBR Green Supermix protocol (Bio-Rad Laboratories). PCR amplification was carried out in 15 µL reaction volume containing 25 ng of cDNA, 1x iQ SYBR Green Supermix, and 250 nM gene specific sense and anti-sense primers. Reactions were run and analyzed on a Bio-Rad iQ5 real-time PCR detection system (IQ5 software version 3.1; Bio-Rad Laboratories) together with reference genes 18SRNA and β-actin. Cycling parameters were determined and analyzed using the Pfaffl modification of the ΔΔCt equation with taking accounts to the efficiency of the reaction [21,22]. The primers for PCR were designed using software Beacon Designer Version 7.9 (Premier Biosoft International, Palo Alto, CA, USA). Nucleotide BLAST was performed to check the specificity of the sequences. Melting curve analysis and agarose gel electrophoresis were carried out to asses templates products. The list of the primers description and sequences was described in Table S1 in File S1.

### Immunofluorescence and Western Blot analysis

The immunofluorescence (IF) analysis was performed on the paraffinated tissues section used for histological analysis. Tissue microarray (TMA) of paired tumor-cirrhosis and normal control, each 1 mm diameter and 4 µm thick, was prepared by microdissection and subjected to IF staining for CD90. To avoid technical variation, all sections were placed on a same TMA slide glass. After de-paraffinization with xylene and rehydration with gradual concentration of ethanol, antigen retrieval was performed by microwave heat in 10 mM sodium citrate pH 6.0. IF staining was quantified by fluorescence area per total area using software ImageJ (National Institute of Health, Bethesda, MA, USA). Three photos per sample were taken with similar exposure condition. For double immunostaining on HCC tissue, anti human CD90-FITC, smooth muscle actin (αSMA) and/or vimentin (VIM)-PE antibodies were added and nucleus was stained using Hoechst 33342 (Sigma-Aldrich). The autofluorescence background of paraffin tissues on single and double staining was quenched by combination of photobleaching and Sudan Black B dye [23]. Single immunostaining and IgG isotype control were also carried out. Proteins positivity was observed by using a fluorescence microscope Leica DM2000 (Leica Camera AG, Solms, Germany).

The CD90 protein analysis was performed by Western Blot (WB) on total protein extract obtained from paired tumoral and cirrhotic tissues of HCC (n=14), CC (n=6), HB (n=10), and normal CTRL (n=6). The protein was extracted from the same samples used for mRNA analysis. Proteins (40 µg) were size-separated by SDS–PAGE on 12% polyacrylamide gel. In the case of JHH-6 cells, the protein expressions of the ATP-Binding Cassette transporters (ABC transporters) ABCB1 (MDR1/multidrug resistance protein 1, ABCC1 (MRP1/multidrug resistance-associated protein 1), and ABCG2 (BCRP/breast cancer resistance protein) were also performed by using 30 µg of membrane protein extract on 10% polyacrilamide gel. The isolation of membrane protein was performed as described by Paulusma [24]. Electro-transferred gel onto PVDF membrane was immunoblotted with the appropriate antibody, as described in Table S2 in File S1. Actin and ATP1A1 were used as housekeeping protein for total and membrane protein extracts, respectively. The peroxidase reaction was obtained by exposure of membrane in the ECL-Plus WB detection system solutions (ECL Plus Western Blotting Detection Reagents, GE-Healthcare Bio-Sciences, Italy). Protein quantification was performed after densitometric analysis of bands CD90 *vs.* actin in each sample.

### Cells separation by magnetic sorting

Separation of CD90-positive cells from JHH-6 cell line was performed using MACS magnetic cell sorting (Miltenyi Biotec GmbH, Bergisch Gladbach, Germany) according to manufacturer’s instruction. A total of 20 millions cells were incubated with CD90-FITC and then were bound with anti-FITC microbeads. After washing, the cells were subjected to magnetic separation. The purity of cells separation was directly checked by flow cytometry and confirmed by real time PCR and WB.

### Flow cytometry

The presence of CD90 protein in JHH-6 cells was detected by flow cytometry. At least two million cells/ml were incubated with CD90-FITC (Stem Cells Technology, Vancouver, Canada) according to manufacturer’s instruction. Flow cytometry analysis was performed immediately using FACSCalibur flow cytometer (Becton Dickinson, NJ, USA). A total of 10,000 events were analyzed per sample.

### Functional test of CD90^+^ cells

#### Growth curve

JHH-6 subpopulations CD90^+^ and CD90^-^ cells were plated in a 24-well plate at an initial concentration of 5,000 cell/ml. Cells growth of six replicates was measured with the MTT ((3-(4,5-dimethylthiazol-2-yl)-2,5-diphenyltetrazolium bromide) dye reduction assay every two days. The absorbance of each well was read on a microplate reader (Beckman Coulter LD 400C, Brea, CA, USA) at 562 nm wavelength. The data represented the mean ± SD of three independent experiments.

#### 3D anchorage-independent matrigel assay

JHH-6 subpopulations CD90^+^ and CD90^-^ cells were grown in low density 5,000 cells/ml in 33% matrigel (BD Biosciences, Buccinasco, Italy) in serum free media. Cells colony diameter in 3D matrix was recorded every two days and measured using software ImageJ, counting 20-100 colonies in each time points. The data represented the mean ± SD of three independent experiments.

#### Response to Doxorubicin

The cytotoxic effect of doxorubicin hydrochloride (DOX) (Sigma-Aldrich, St Louis, MO, USA) was assessed by MTT dye reduction assay. JHH-6 subpopulations CD90^+^ and CD90^-^ cells were seeded at an initial concentration 500,000 cell/ml. DOX was given to the cells in a concentration range between 0 and 40 µg/ml for 24 hours. The absorbance of the untreated controls was taken as 100% survival. The expression of ABC-transporters ABCB1, ABCC1, and ABCG2 were analyzed by RT-qPCR and WB as described above. The data represented the mean ± SD of minimum three independent experiments.

#### Animal study

For animal study, one million of each JHH-6 subpopulations CD90^+^ and CD90^-^ in cold PBS was injected orthotopically in the liver of NOD/SCID mice. Duplicates were performed for each subpopulations. The viability test of the residual cells in PBS checked by tryphan blue staining showed viability higher than 90%. The xenografts were observed for four months after injection. RT-qPCR analysis was performed to detect human and mouse genes in the liver and lung tissues of xenograft, each minimum in duplicates.

### Statistical analysis

Statistical analysis was performed using software InStat Version 3.05 (GraphPad Software, Inc., La Jolla, CA, USA). For human tissues samples, continuous variables of CD90 mRNA distribution by RT-qPCR and protein expression by WB and IF were calculated using one-way Anova with Bonferroni post-test following normality test and values were represented as mean ± SD. Associations between continuous variables (CD90 mRNA levels) and clinicopathological variables were evaluated using Mann–Whitney tests for two groups (grade 1 & 2 *vs.* 3 & 4; serum AFP <100 *vs.* >100 ng/ml, CTP A *vs.* BC and MELD score <9 *vs.* >9). For cell line JHH-6, data was represented as mean ± SD, unpaired students’ *t* test was performed to compare CD90^+^ and CD90^-^ cells. Statistical significance was set to p<0.05.

## Results

### The up-regulation of CD90 in HCC and other tumors

**Figure 1:**
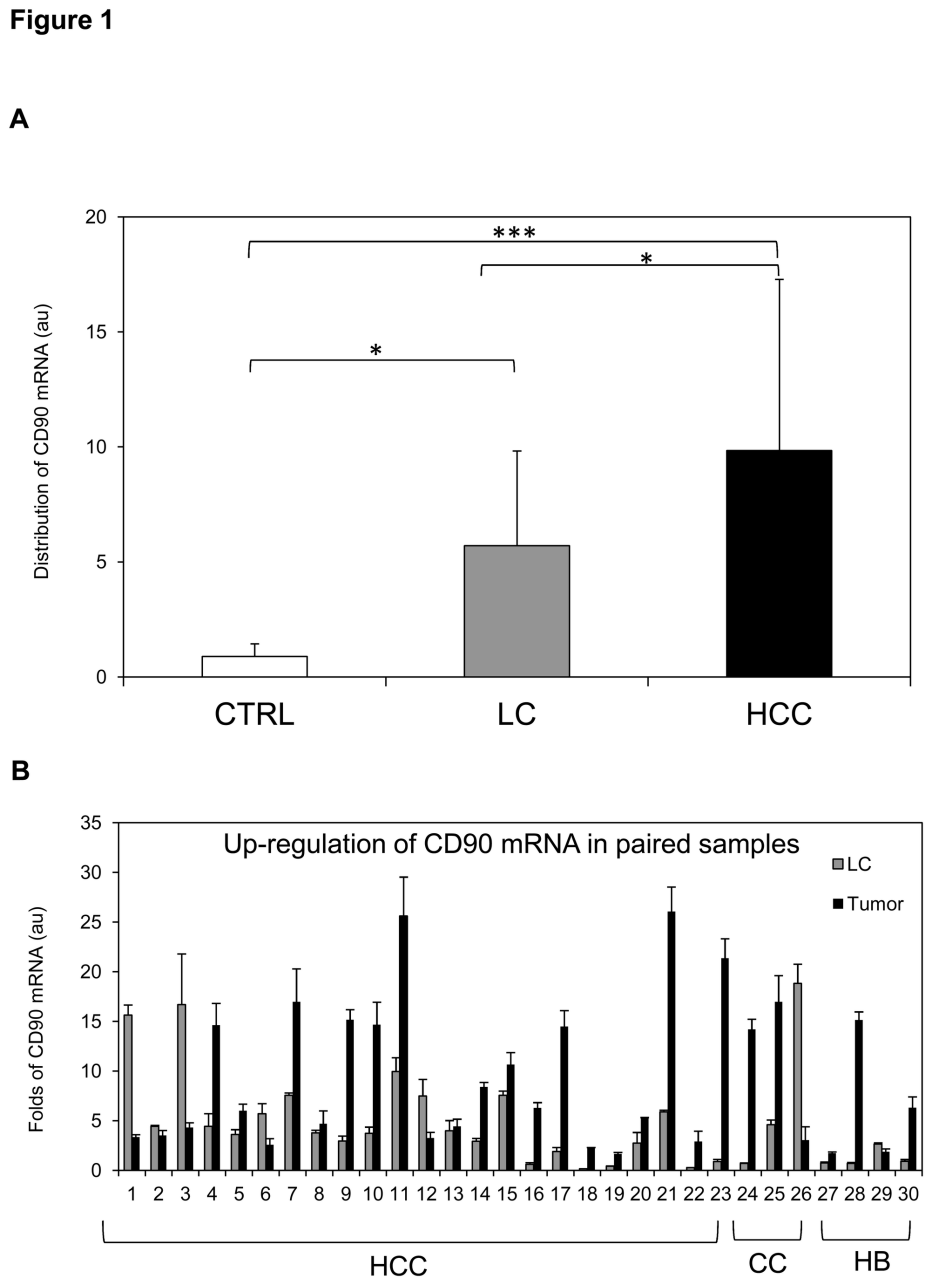
The mRNA expression of CD90/Thy-1 in primary liver cancer by RT-qPCR analysis. **A**. CD90 distribution in normal liver CTRL (n=12), liver cirrhosis LC of HCC (n=26) and HCC (n=25). Statistical analysis was performed using one-way ANOVA with Bonferroni post-test (*p<0.05, ***p<0.001). Data represented mean ± SD. **B**. CD90 gene up-regulation in paired individuals of HCC (1-23), cholangiocarcinoma (CC, 24-26), and hepatoblastoma (HB, 27-30). CD90 mRNA relative expression was normalized to reference genes 18SRNA and β-actin; the expression of a normal sample was considered as 1.00 au.

The CD90 mRNA in human tissue specimens was analyzed by RT-qPCR and expressed in arbitrary unit (au) with a normal liver considered as 1.00 au. As shown in Figure 1A, a significant increase of CD90 was observed in HCC as compared either to the liver cirrhosis (LC) or normal CTRL (p<0.05 and p<0.001, respectively). The mean ± SD values were 9.84 ± 7.44 for HCC and 5.71 ± 4.12 for LC; 0.89 ± 0.55 was the value for CTRL (p<0.05 and p<0.001 compared to LC and HCC, respectively). When the CD90 expression was compared in paired samples of HCC and LC, as shown in Figure 1B, gene up-regulation was observed in 18/23 (78%) cases, 2/3 (67%) of CC, and 3/4 (75%) of HB, although the extent of up-regulation was quite variable. In HCC, high CD90 mRNA expression was related to serum AFP level less than 100 ng/ml, but not to Edmonson-Steiner histological grade, CTP and MELD score.

**Figure 2:**
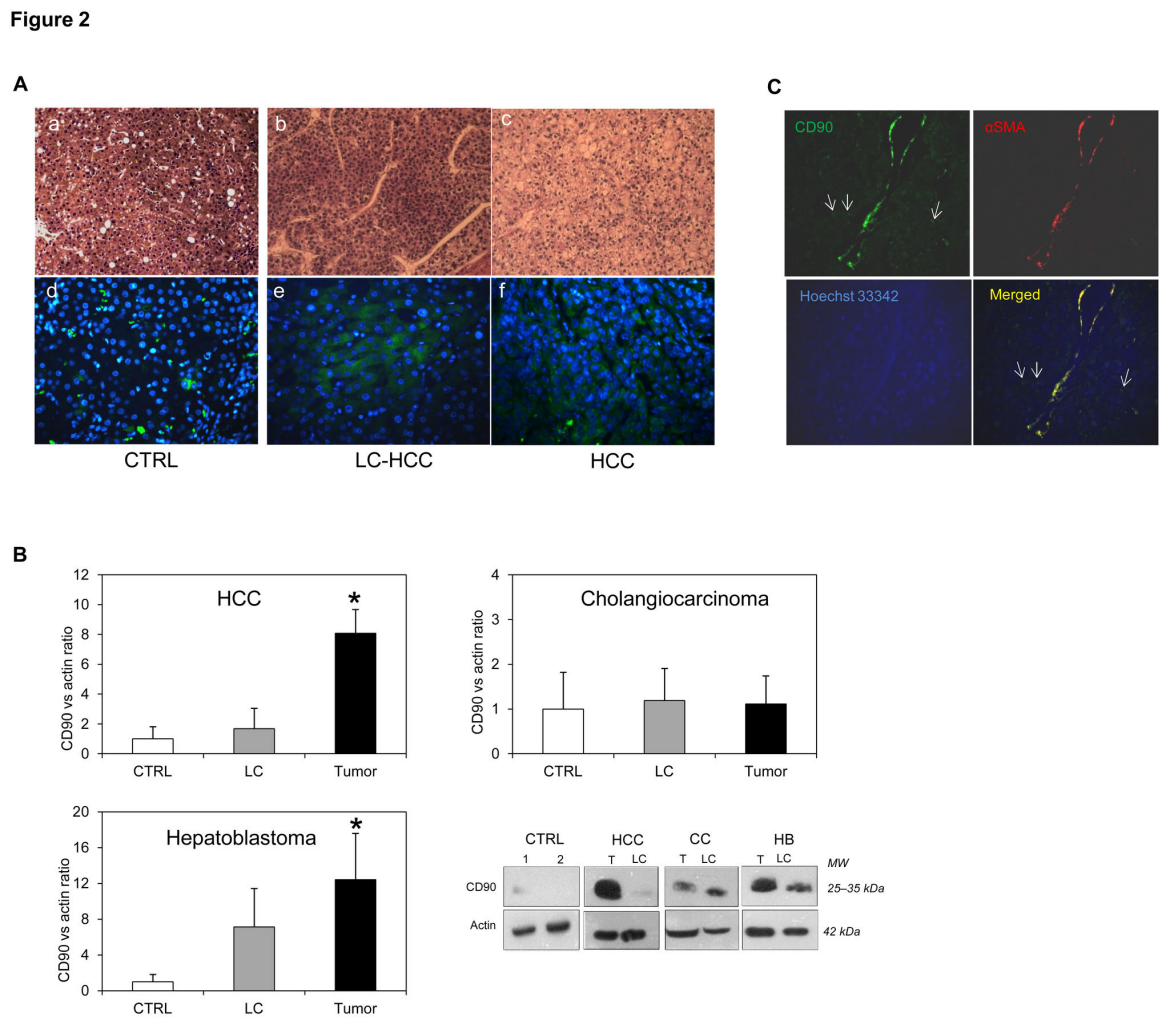
The protein analysis of CD90/Thy-1 in primary liver cancers. **A**. Histological hematoxylin-eosin analysis and CD90 protein distribution in microdissected tissues of normal CTRL (*a*,*d*), liver cirrhotic LC of HCC (*b*,*e*) and tumoral tissue HCC (*c*,*f*). Magnification: objective 20X (*a*-*c*), 40x (*d*-*f*); CD90-FITC: green, Hoechst 33342: blue. **B**. Western Blot analysis represented mean ± SD values of CD90 protein expression in LC and tumoral tissues of HCC (n=14), cholangiocarcinoma (CC, n=6), and hepatoblastoma (HB, n=10) compared to normal CTRL (n=6). Protein quantification was performed using densitometric analysis of bands CD90 *vs*. actin in each sample. Statistical analysis was performed using one-way Anova with Bonferroni post-test (*p<0.05 compared to CTRL). *Lower* *right* *panel*: representative blot of each paired tissues compared to CTRL. **C**. The co-expression of CD90 with αSMA in tumoral tissue of HCC. CD90 protein (green), αSMA (red), and nucleus (Hoechst 33342, blue). Arrows indicated CD90^+^SMA^-^ cells. Magnification: objective 20X.

To investigate if the increased CD90 gene up-regulation was associated to an increased amount of protein, the CD90 protein content was assessed by IF in paired tissues on a TMA microdissected slide of 5 HCC, 2 CC, and 2 CTRL. Since as reported previously almost all liver cells showed background signal in basal conditions, a strong signal against background was considered as positive [15]. As shown in Figure 2A, CD90 positive cells were markedly more present in HCC as compared to LC and CTRL in all HCC samples examined. The percentage of CD90 positive area (mean ± SD) was quantified as 2.8 ± 1.5% for CTRL, 12.4 ± 5.1% for LC, and 21.1 ± 6.6% for HCC. Furthermore, WB analysis was performed in paired samples of randomly selected 14 HCC, 6 CC, and 10 HB, together with 6 controls. mRNA analysis was also performed in all samples where the protein content was measured. In Figure 2B, WB analysis confirmed a significant up-regulation of CD90 protein in the tumoral tissue of HCC which was around eight-fold higher that in CTRL (p<0.05). A similar, though less evident (p<0.05) up-regulation was also noticed in HB samples. In contrast, the difference was not significant in CC when compared both the LC and CTRL.

### Tumor promoting properties of CD90^+^ cells

**Figure 3:**
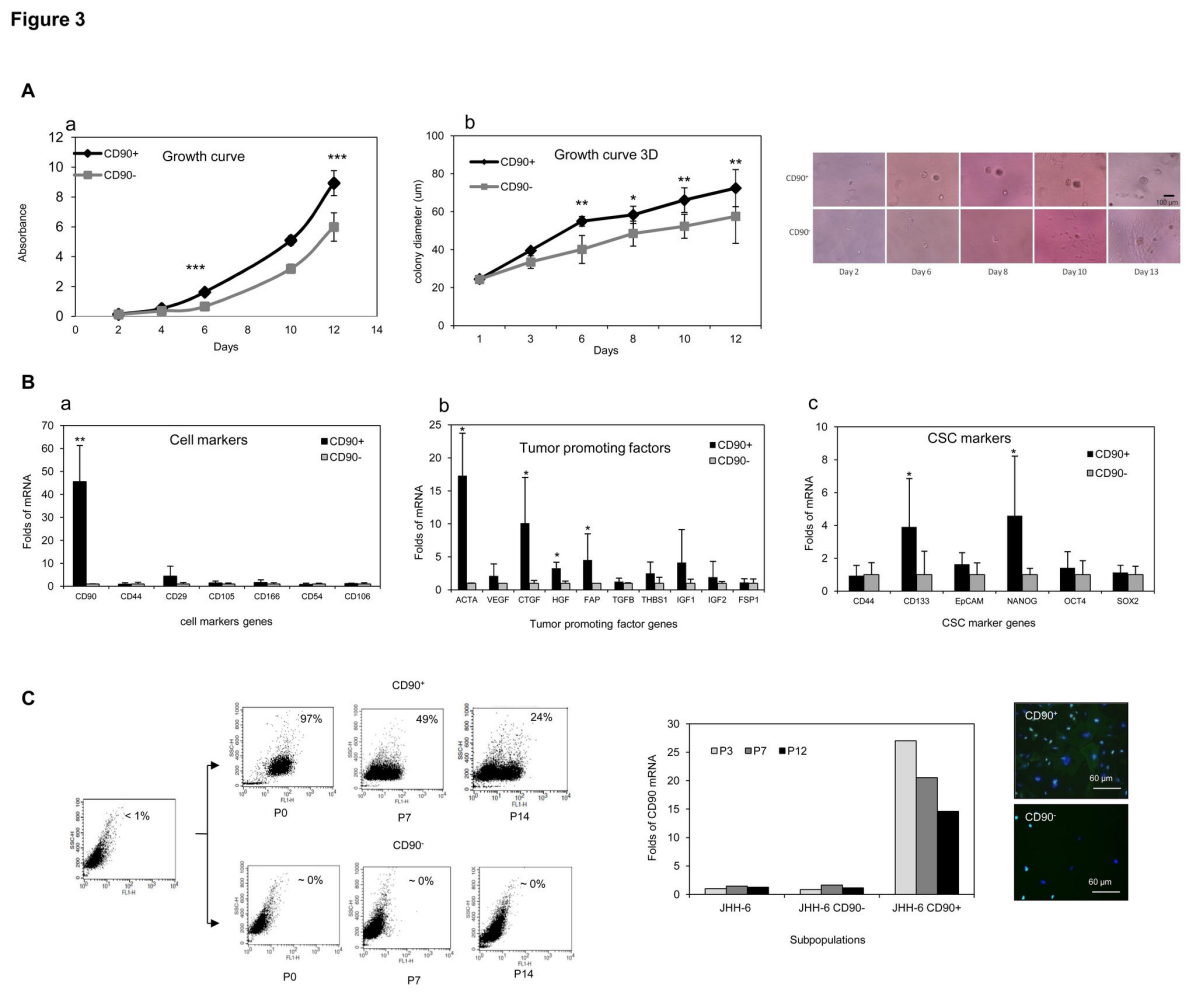
The growth capacity of the JHH-6 subpopulations CD90^+^ and CD90^-^. **A**. Growth curve after 12 days subculture of attached cells (*a*) and 3D colony in matrigel (*b*). **B**. mRNA analysis of cells markers (*a*), tumor promoting factors (*b*), and CSC markers genes (*c*). mRNA relative expression was normalized to reference genes 18SRNA and β-actin; the expression of CD90^-^ cells was considered as 1.00 (Students’ *t* test *p<0.05, ***p<0.001 compared to CD90^-^). **C**. The alteration of CD90^+^ cells phenotypes during subculture. *Upper* *panel*: FACS analysis of fresh-purified JHH-6 subpopulations CD90^+^ and CD90^-^ during subculture (passage 1-15). The number in each plots represented CD90^+^ positivity. *Lower* *left* *panel*: CD90 mRNA analysis of subpopulations CD90^+^ and CD90^-^ together with unsorted JHH-6 cells during subculture. *Lower* *right* *panel*: IF analysis of subpopulations CD90^+^ and CD90^-^. Magnification: objective 40X.

JHH-6 cells were separated in CD90^+^ and CD90^-^ subpopulation using magnetic sorting and the purity of cells separation was directly confirmed by RT-qPCR, WB, flow cytometry and IF (data not shown). The growth curve of both subpopulations showed that CD90^+^ cells had a significant higher capacity to proliferate compared to CD90^-^ cells (p<0.001), starting six days after plating. This capacity was also observed in anchorage-independent manner in 3D colony growth, CD90^+^ cells had higher capacity to growth independently compared to CD90^-^ cells, demonstrated by larger diameter of the colonies (p<0.05) (Figure 3A).

The gene expression analysis of other cell markers (CD44, CD29, CD105, CD166, CD54, CD106 and OCT4) in these freshly separated two subpopulations showed that apart from the CD90 (p<0.01), no significant difference in other cell markers was found. Further examination on several genes involved in the cancer growth showed that the expressions of hepatocyte growth factor (HGF), fibroblast associated protein (FAP) and alpha smooth muscle actin 2 (ACTA2) were significantly increased in CD90^+^ subpopulation (p<0.05). On the contrary, no variation in the expression of other factors as of connective tissue growth factor (CTGF), vascular endothelial growth factor (VEGF), transforming growth factor beta 1 (TGFβ1), thrombospondin (THBS), insulin growth factor 1 (IGF1), and insulin growth factor 2 (IGF2) was observed. In relation with CSC, the CD90^+^ cells expressed significantly higher mRNA level of CSC marker CD133 and pluripotency factor NANOG (Figure 3B).

### Loss of CD90 phenotype

After cell sorting and assessment of their purity, cell phenotype from both CD90 positive and negative fractions was observed along subsequent subcultures by flow cytometer and RT-qPCR. It was found that CD90^+^ subpopulation lost its CD90 phenotype into both CD90^+^ and CD90^-^ phenotypes while the CD90^-^ cells harbored only CD90^-^ cells. At the 21^st^ passage after sorting, the original CD90^+^ subpopulation was composed by 20% CD90^+^ and 80% CD90^-^ cells. On the contrary, the CD90^-^ subpopulation persisted to be 100% CD90^-^ cells. The change of CD90 phenotypical marker was confirmed by gene quantification using RT-qPCR which showed a decrease of CD90 expression along subculture in CD90^+^; unsorted JHH-6 showed a steady expression (Figure 3C).

### Expression of drug-resistance transporter in CD90^+^ cells

**Figure 4:**
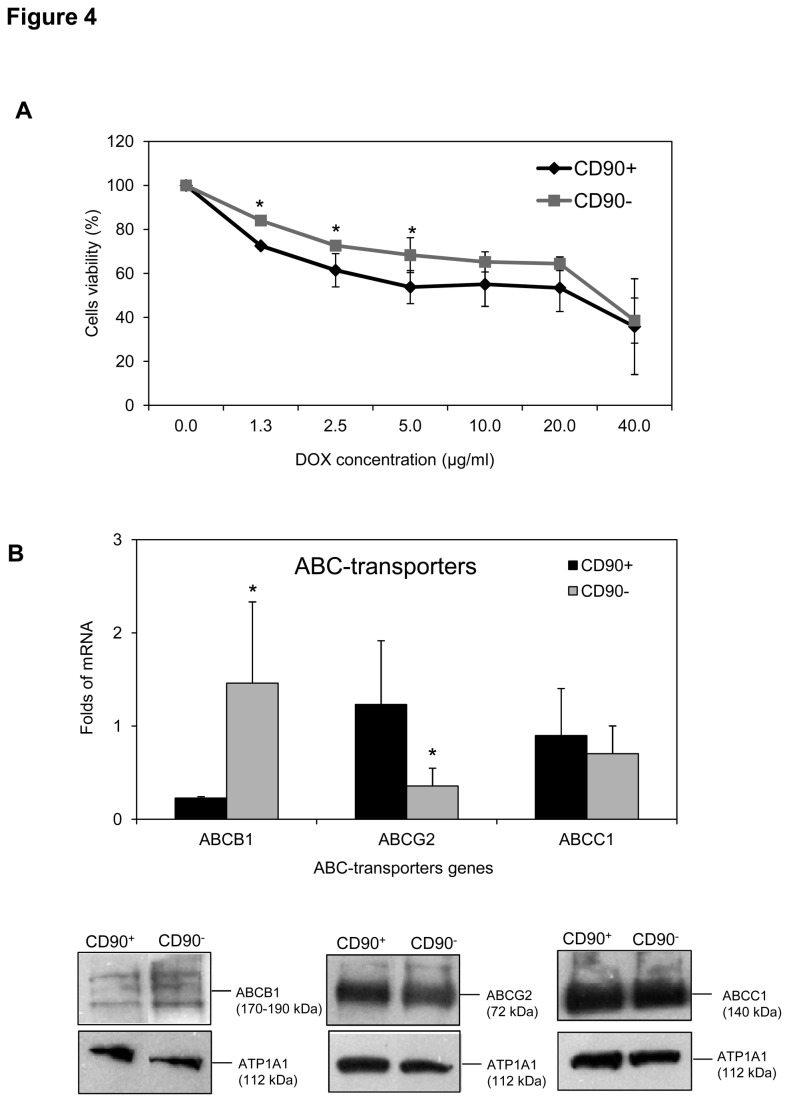
The potency of DOX resistance of the JHH-6 subpopulations CD90^+^ and CD90^-^. **A**. Cells viability against DOX (0-40 µg/ml) for 24 hours assessed by MTT assay. Non-treated cells were considered as 100% viability (Students’ t test *p<0.05). The data represented the mean ± SD of three independent experiments. **B**. The mRNA and protein expression of DOX-related ABC transporters ABCB1, ABCCC1, and ABCG2 genes (Students’ *t* test *p<0.05 compared to CD90^-^). *Low* *panel*: WB analysis of each proteins ABCG2 (72 kDa), ABCB1 (170-190 kDa), and ABCC1 (140 kDa) together with ATP1A1 membrane protein (112 KDa).

As shown in Figure 4A, 24 hours after exposure to a DOX at a concentration between 0 and 40 µg/ml, a significant difference was noticed in DOX treatment of 1.3 to 5.0 µg/ml (p<0.05). The analysis of the gene expression of three ABC-transporters ABCB1, ABCC1, and ABCG2 mainly involved in DOX-resistance showed a higher expression of ABCG2 and an inversely lower ABCB1 expression in subpopulation CD90^+^ (p<0.05). No difference was found in ABCC1 expression. The modulation in RNA expression was supported by WB analysis on all three genes (Figure 4B).

## Discussion

The PLC, especially HCC, is one of the most common neoplasms in the world and is frequently associated with a chronic liver disease such as cirrhosis. In this study, we describe a clear increase of CD90 moving from healthy liver to cirrhosis and HCC, ideally representing the natural course of the hepatocarcinogenesis. This result expands a previous report which showed that CD90 expression was higher in HCC compared to normal tissues [15].

Based on double-staining with CAFs markers αSMA and VIM [25], we observed that in HCC, the majority of CD90^+^ cells were also positive for αSMA, and they were distributed majorly in the stromal component. It indicated that the up-regulation of CD90 may be contributed by the activation of myofibroblasts and the presence of CAFs in LC and HCC. The activation of these cells was also shown by the increase of genes ACTA2 and VIM, even though we did not see any linear correlations between two genes (Figure S1 and Figure S2 in File S1). Interestingly, we also observed a small number of CD90^+^ cells that were negative for αSMA and VIM, suggesting that there was a different population of CD90^+^ cells in the cancer (Figure 2C and Figure S1 in File S1).

In diseased liver, CD90 was expressed in hepatic stem cells, hepatic fibroblasts, myofibroblasts, and tumor stroma (CAFs), and small percentage of CSC [8,9,14–16,26]. The origin of the myofibroblasts and CAFs might be derived from the epithelial mesenchymal transition (EMT), resident fibroblasts, or bone marrow derived stem cells. It has been demonstrated that multipotent adult stem cells with CAFs properties was found not only in HCC, but also in cirrhotic liver, supporting the evidence that the CAFs could be originated from resident progenitor cells [27].

Based on clinical data, serum AFP level higher than 100 ng/ml was found in the group with low CD90 mRNA expression. This data is in accordance with previous report showing a lower AFP RNA level in CSC phenotype CD90^+^CD45^-^ in HCC [16]. The two samples with the highest expression in LC region were obtained from HBV positive patients, in line with a recent report demonstrating that CD90 is associated with HBV infection [15]. No significant correlations was found for etiology, CTP and MELD score, and histological grade. Unfortunately due to small size of samples, statistical analysis on the association of CD90 expression to tumor recurrence and time of survival could not be performed. Of notice is the observation that the up-regulation of CD90 is not an exclusive pattern in HCC as a similar behavior was also found in samples of other liver neoplasm such as HB, suggesting that CD90 may be involved the process of the development of hepatic tumors.

Since the CD90 is a marker for different cell types, we explored its function in HCC by using an *in vitro* model using JHH-6 HCC cell line. Different with report from Yang et al. [16] we could not isolate CD90^+^ cells from HuH-7 and HepG2, in concordant with recent report from Yamashita et al. [18]. JHH-6 is an aggressive HCC cell line with undifferentiated morphology [28], in basal condition this cell line expresses CD90 mRNA in less than 1% cells as measured by FACS. During subcultures, the CD90^+^ cells of the JHH-6 line lost its phenotype. This observation may be at the basis of the small percentage of CD90^+^ cells in the original JHH-6 line in spite of their high growth rate. This capacity had been reported for the ABCG2 protein in HuH-7 and PLC cell lines obtained from HCC [29]. This data shows that CD90^+^ cells might be placed higher than CD90^-^ cells in hierarchy level of cancer, and might be one of cell types responsible of generating cancer heterogeneity. Nevertheless, the loss of CD90 protein of the cells can be also caused by protein release into growth media.

In JHH-6 CD90^+^ and CD90^-^ cells, the expression of other cell markers genes (CD44, CD29, CD105, CD166, CD54, and CD106) showed that apart from CD90, no significant difference in other cell markers was observed. CD90^+^ cells have a higher proliferation capacity compared to its CD90^-^ counterpart, also noticed in 3D clonogenic assay. This capacity may be related with the higher expression of HGF, FAP and ACTA2. The expression of TGFβ1, VEGF, THBS1, CTGF, IGF1, IGF2, and FSP1 were variably and inconsistently increased. Further analysis of EMT related molecules demonstrated that the CD90^+^ cells were not originated from this process. Despite of higher expression of SNAIL, no significant differences was observed in the direction of EMT process (Figure S3 and Figure S4 in File S1).

The higher CD90^+^ cells proliferation ability and higher tumor promoting capacity observed *in vitro* correlates with the *in vivo* data where CD90^+^ molecule was found to be increased during the progression of HCC. The involvement of CD90 cells in tumor progression was also reported in CAFs obtained from prostate cancer in which the CD90 could serve as cancer biomarker [12,30].

The exposure of the cell to DOX is associated with the regulation of several ABC-transporters protein, in particular ABCB1, ABCC1, and ABCG2, specific transporters of DOX. Interestingly, the CD90^+^ cells showed a higher expression of ABCG2/BCRP and lower ABCB1/MDR1 as compared to CD90^-^ cells. In contrast, the expression of both genes was inversely expressed in CD90^-^ cells suggesting a complimentary effect of both genes in overcoming the toxicity of DOX. The relation of ABCG2 with progenitor cells and differentiation had been reported in undifferentiated human embryonic cells [31] and hematopoietic system [32]. Accordingly, the functional activity of this transporter to target CD90 cells in drug therapy must be considered.

Since CD90 had been proposed as a single marker for the CSC [16], we checked also others CSC markers in these subpopulations. Interestingly, in JHH-6 CD90^+^ subpopulation, a higher expression of CSC marker CD133 and NANOG, was observed. This data is concordant with a previous study which showed that CD90^+^ cells in the tumor tissues concomitantly express stem cell markers such as CD133 [16].

In addition, we performed a xenograft model. JHH-6 cells did not induced tumor after subcutaneous injection to nude mice. By intrahepatic injection CD90^+^ and CD90^-^ JHH-6 cells to NOD/SCID mouse, even though no visible HCC nodules were noticed 4 months after injection, we observed that murine Afp mRNA expression in the liver of mouse injected with CD90^+^ was around 3 times higher compared to CD90^-^, meanwhile the albumin mRNA in lung was strikingly up-regulated compared to both CD90^-^ and control mice (Figure S5 in File S1). However, the human genes of CD90, AFP, and ALB were not or very low expressed. The negativity of tumor nodule is in agreement with previous data by Murakami et al. that JHH-6 did not give clear tumor nodule [33]. This failure of CD90^+^ cells from JHH-6 cells to induce tumor is different with what had been reported by Yang et al. with CSC CD90^+^ cells from HuH-7, HepG2, MHCC97L [16]. However in a more recent paper it has been reported that the tumorigenic CSC CD90^+^ cells might appear in the later stages of hepatocarcinogenesis and only identified in HBV related HCC [18]. Since JHH-6 cell line was derived from HBV(-) HCC, this might be the explanation of later/differences on the tumorigenecity of CD90^+^ cells.

In spite of the high variation of the role of CD90 in cancer, this study highlights the importance of CD90 molecule in the development of primary cancer in liver, in particular in HCC. However, due to the diversity of the identity and cellular function of CD90^+^ cells in tumor, other molecular markers would be helpful to further characterize the cancer and develop phenotypic target in HCC therapy.

## Supporting Information

File S1 **Combined file.** Table S1, List of primers. TS=this study. Table S2, List of antibodies. Figure S1, Expression of CD90, αSMA and VIM in a HCC by IF. Objective magnification: 10X (A) 40X (B). Figure S2, mRNAexpression of CD90, VIM, and ACTA2 in primary liver cancer by RT-qPCR analysis. A. VIM and ACTA2 distribution in normal, liver cirrhosis LC of HCC and HCC. Boxes indicate the percentiles, lines the median au value for each groups (*p<0.05, **p<0.005). B. The comparison of CD90, VIM, and ACTA2 genes up-regulation in paired individuals of HCC. mRNA relative expression was normalized to reference genes 18SRNA and β-actin; the expression of LC of HCC-20 was considered as 1.00 au. Figure S3, Presence of antigen Vimentin, CD44, and CD133 in JHH-6 subpopulations CD90^+^ and CD90^-^ by FACS analysis. - control – positivity. Figure S4, mRNA analysis of epithelial mesenchymal transition (EMT) cells markers. mRNA relative expression was normalized to reference genes 18SRNA and β-actin; the expression of CD90^-^ cells was considered as 1.00 (Students’ *t* test *p<0.05 compared to CD90^-^). Figure S5, Murine mRNA analysis of liver and lung tissues of xenograft in NOD/SCID mice. Afp=alpha fetoprotein, Alb= albumin. Wild type = CTRL mice, XG JHH-6 CD90+ = xenograft injected with JHH-6 CD90^+^, XG JHH-6 CD90- = xenograft injected with JHH-6 CD90^-^. mRNA relative expression was normalized to reference genes β-actin. Reference list of the File S1.
(PDF)
