# Supplementary material for: The Expression of CD90/Thy-1 in Hepatocellular Carcinoma: An In Vivo and In Vitro Study
Source: PLoS One. 2013 Oct 8;8(10):e76830. doi: 10.1371/journal.pone.0076830 (PMC3792890; doi:10.1371/journal.pone.0076830)
Supplement: File S1 — Combined file. Table S1, List of primers. TS=this study. Table S2, List of antibodies. Figure S1, Expression of CD90, αSMA and VIM in a HCC by IF. Objective magnification: 10X (A) 40X (B). Figure S2, mRNAexpression of CD90, VIM, and ACTA2 in primary liver cancer by RT-qPCR analysis. A. VIM and ACTA2 distribution in normal, liver cirrhosis LC of HCC and HCC. Boxes indicate the percentiles, lines the median au value for each groups (*p<0.05, **p<0.005). B. The comparison of CD90, VIM, and ACTA2 genes up-regulation in paired individuals of HCC. mRNA relative expression was normalized to reference genes 18SRNA and β-actin; the expression of LC of HCC-20 was considered as 1.00 au. Figure S3, Presence of antigen Vimentin, CD44, and CD133 in JHH-6 subpopulations CD90+ and CD90- by FACS analysis. - control – positivity. Figure S4, mRNA analysis of epithelial mesenchymal transition (EMT) cells markers. mRNA relative expression was normalized to reference genes 18SRNA and β-actin; the expression of CD90- cells was considered as 1.00 (Students’ t test *p<0.05 compared to CD90-). Figure S5, Murine mRNA analysis of liver and lung tissues of xenograft in NOD/SCID mice. Afp=alpha fetoprotein, Alb= albumin. Wild type = CTRL mice, XG JHH-6 CD90+ = xenograft injected with JHH-6 CD90+, XG JHH-6 CD90- = xenograft injected with JHH-6 CD90-. mRNA relative expression was normalized to reference genes β-actin. Reference list of the File S1. (PDF) [file pone.0076830.s001.pdf]

FILE S1. SUPPLEMENTAL DATA

Table S1

Gene	Acc. No.	Sequence F (5’ → 3’)	Sequence R (5’ → 3’)	Ref.
18SRNA	NR_003286	TAACCCGTTGAACCCCATT	CCATCCAATCGGTAGTAGCG	[S1]
Beta-Actin	NM_001101	CGCCGCCAGCTCACCATG	CACGATGGAGGGGAAGACGG	TS
CD90	NM_006288	AGAGACTTGGATGAGGAG	CTGAGAATGCTGGAGATG	TS
CD29	NM_002211	GCCTTGGTGTCTGTGCTGAG	AGTCGTCAACATCCTTCTCCTTAC	TS
CD44	NM_000610	CTCATACCAGCCATCCAATG	GAGTCCATATCCATCCTTCTTC	TS
CD105	NM_001114753	CTTCCTCCTCCACTTCTAC	GGACTTCCTGGTCTTGAG	TS
CD166	NM_001627	GAAGGAGGAGGAATATGGAATC	GTCAAGTCGGCAAGGTATG	TS
CD54	NM_000201	GCTCAAGTGTCTAAAGGATGG	TATGACTGCGGCTGCTAC	TS
CD106	NM_001078	GACCACATCTACGCTGAC	GCAACTGAACACTTGACTG	TS
THBS	NM_003246	ACCAACCGCATTCCAGAGTC	CCGCACAGCATCCACCAG	TS
VEGF	NM_001171630	GTGAGGCGGCGGTGTG	GCAAGGCAAGGCTCCAATG	TS
FAP	NM_004460	TATTCAGAGTAACACAGGATTCA	ACTTCTTGCTTGGAGGATAG	TS
FSP1	NM_019554	CTTCTTCTTTCTTGGTTTGGT	AGCAGTCAGGATCAACAC	TS
ACTA2	NM_001141945	TTGGCTTGGCTTGTCAGG	GCTTTAGGGTCGCTGGAG	TS
TGFB1	NM_000660	GCAACAATTCCTGGCGATACC	CTCCACGGCTCAACCACTG	TS
IGF1	NM_001111284	TCTGAATCTTGGCTGCTGGAG	GGTGTGCTTCTTGACGACTTG	TS
IGF2	NM_000612	CGCTGCTACCGCCATCTC	GTCCCTCTGACTGCTCTGTG	TS
HGF	NM_001010932	AAGGTCAAGGAGAAGGCTACAG	TGCTCGTGAGGATACTGAGAATC	TS

CTGF	NM_001901	AGACCTGTGCCTGCCATTAC	TGTGAATCAGTTCAAGTTCCAGTC	TS
ABCG2	NM_004827	TATAGCTCAGATCATTGTCACAGTC	GTTGGTCGTCAGGAAGAAGAG	TS
ABCC1	NM_004996	GCCAAGAAGGAGGAGACC	AGGAAGATGCTGAGGAAGG	TS
ABCB1	NM_000927	TGCTCAGACAGGATGTGAGTTG	AATTACAGCAAGCCTGGAACC	TS
EpCAM	NM_002354.1	GAATAATAATCGTCAATGCCAGTG	CGCTCTCATCGCAGTCAG	TS
VIM	NM_003380.3	AACTTCTCAGCATCACGATGAC	TTGTAGGAGTGTCGGTTGTTAAG	TS
CD133	NM_006017	CATCTGCTCTCTGCTGAC	AACTTAATCCAACTCCAACC	TS
OCT4	NM_001159542.1	AGCAAAACCCGGAGGAGT	CCACATCGGCCTGTGTATATC	[S2]
SOX2	NM_003106.3	TATTTGAATCAGTCTGCCGAG	ATGTACCTGTTATAAGGATGATATTAGT	[S2]
NANOG	NM_024865.2	ATGCCTCACACGGAGACTGT	AGGGCTGTCCTGAATAAGCA	[S2]
CDH1	NM_004360.3	GGAACTATGAAAAGTGGGCTTG	AAATTGCCAGGCTCAATGAC	[S2]
CDH2	NM_001792.3	GACGGTTCGCCATCCAGAC	TCGATTGGTTTGACCACGG	[S3]
SNAI1	NM_005985.3	CTTCGGCTCCAGGAGAGTC	TTCCCACTGTCCTCATCTGAC	[S2]
SNAI2	NM_003068.4	TGGTTGCTTCAAGGACACAT	GTTGCAGTGAGGGCAAGAA	[S2]

Table S2

Antibody	Clone	MW kDa
CD90/Thy-1	5E10 (Stem Cell Technologies, VA, Canada)	25 – 35
ABCG2/BCRP	BXP53 (Abcam, Cambridge, MA, USA)	72
ABCB1/MDR1	C219 (Abcam, Cambridge, MA, USA)	170 – 190
ACBC1/MRP1	A23 (Enzo Life Sciences, Lausanne, Switzerland)	140
CD133/Prominin 1	AC133 (Miltenyi Biotec GmbH, Bergisch Gladbach, Germany	117
CD44	F10-44-2 (Abcam, Cambridge, MA, USA)	100-250
α smooth muscle actin	1A4 (Dako, Glostrup, Denmark)	42
Vimentin	VI-RE/1 (Abcam, Cambridge, MA, USA)	57
actin	A2066 (Sigma, St. Louis, MO, USA)	42
ATP1A1	464.6 (Abcam, Cambridge, MA, USA)	112
Anti rabbit IgG peroxidase	P0448 (Dako, Glostrup, Denmark)	
Anti rat IgG peroxidase	P0450 (Dako, Glostrup, Denmark)	
Anti mouse IgG peroxidase	P0260 (Dako, Glostrup, Denmark)	
Anti mouse IgG FITC	715-096-150 (Jackson ImmunoResearch Lab., West Grove, PA, USA)	

Figure S1

A

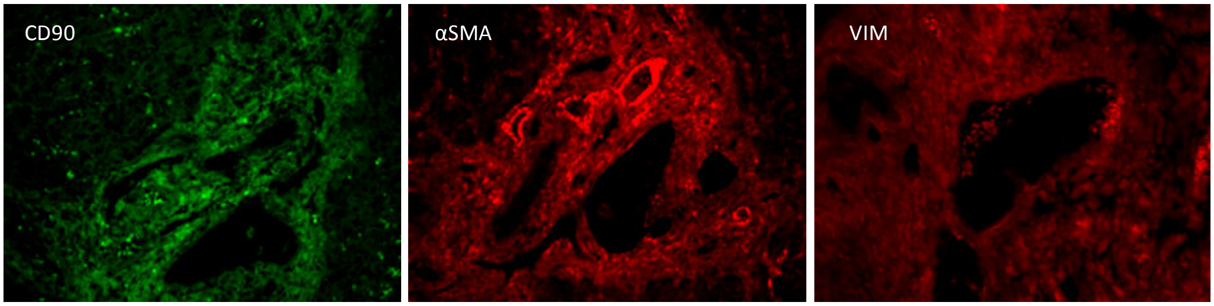

B

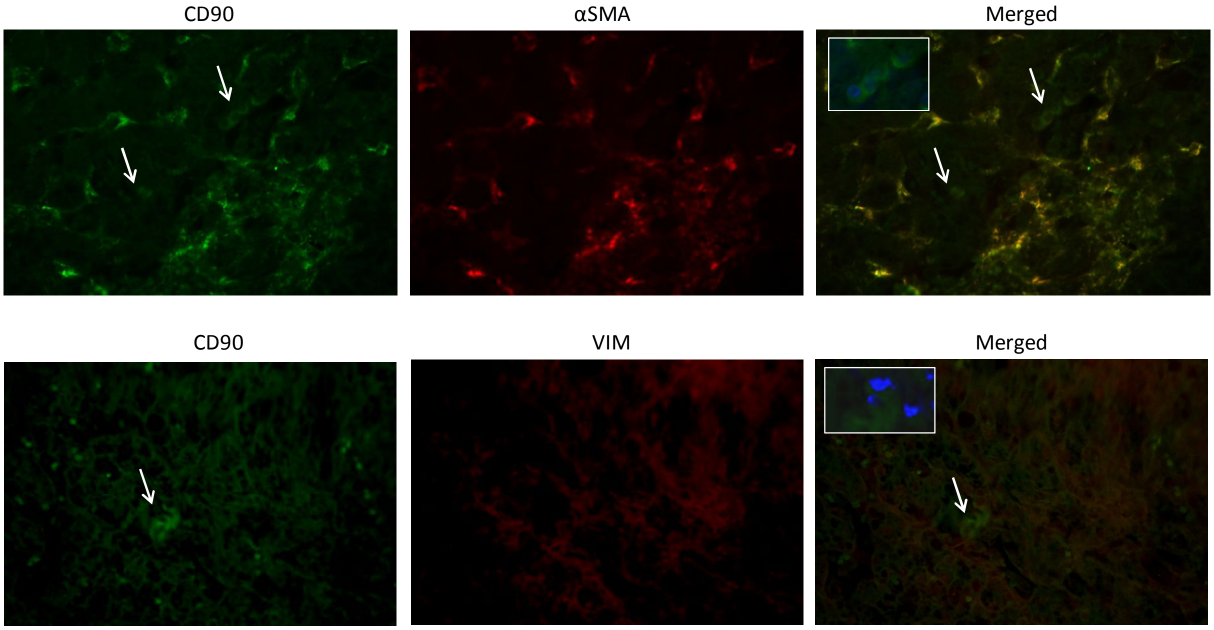

Figure S2

A

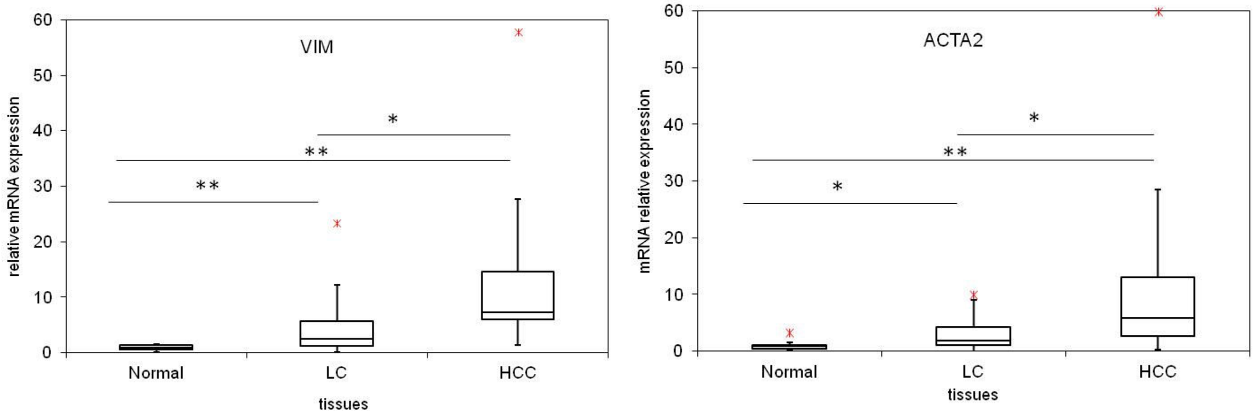
VIM
ACTA2
relative mRNA expression
mRNA relative expression
Normal
LC
HCC
tissues
0
10
20
30
40
50
60
*
**

B

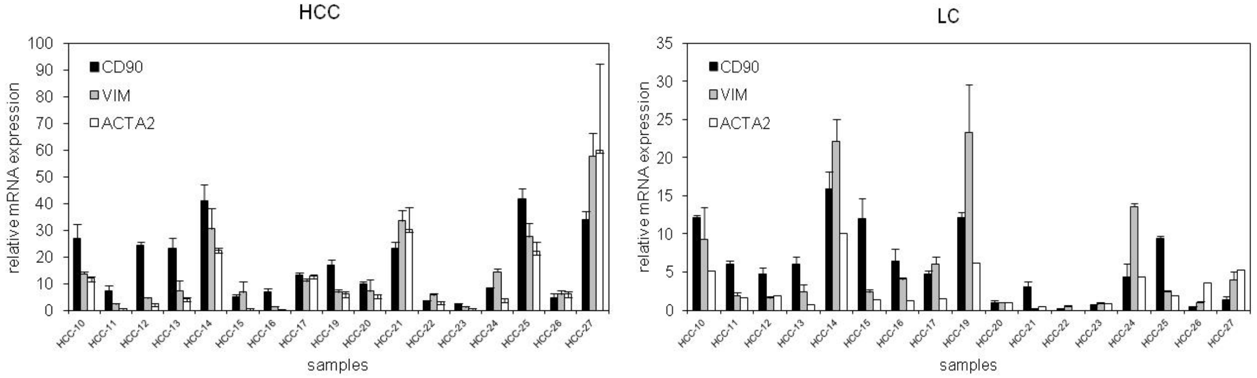
HCC
LC
CD90
VIM
ACTA2
relative mRNA expression
samples
HCC-10
HCC-11
HCC-12
HCC-13
HCC-14
HCC-15
HCC-16
HCC-17
HCC-19
HCC-20
HCC-21
HCC-22
HCC-23
HCC-24
HCC-25
HCC-26
HCC-27

Figure S3

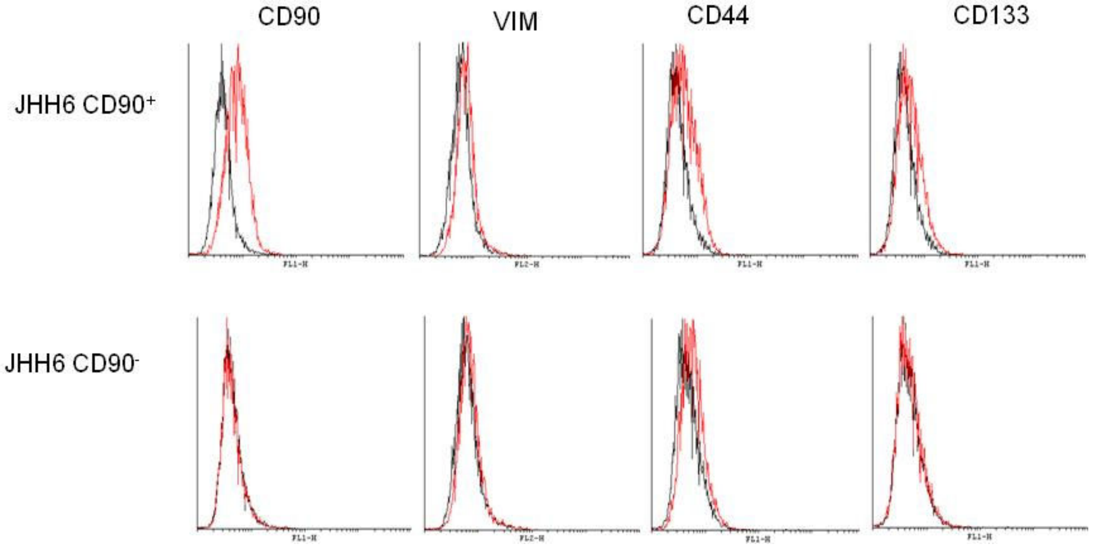

Figure S4

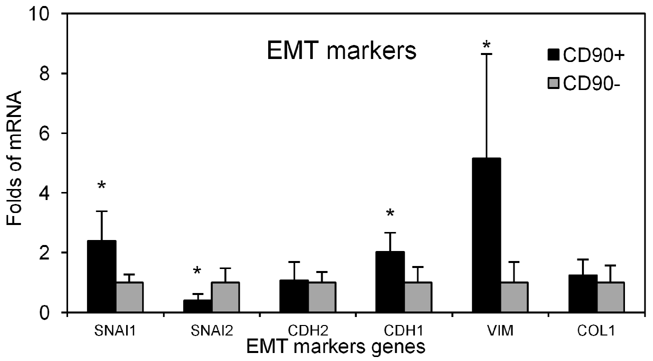

EMT markers
CD90+
CD90-
Folds of mRNA
0
2
4
6
8
10
*
*
*
*
SNAI1
SNAI2
CDH2
CDH1
VIM
COL1
EMT markers genes

Figure S5

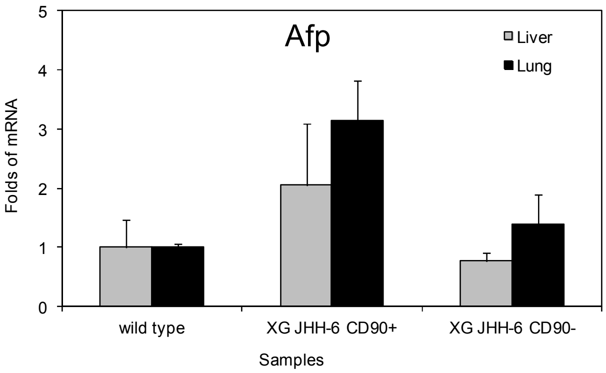
Afp
Liver
Lung
Folds of mRNA
0
1
2
3
4
5
wild type
XG JHH-6 CD90+
XG JHH-6 CD90-
Samples

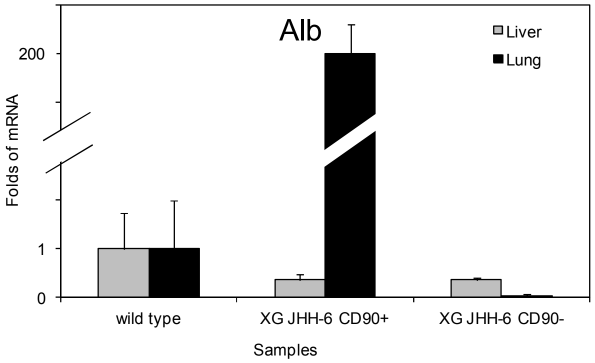
Alb
Liver
Lung
Folds of mRNA
0
1
200
wild type
XG JHH-6 CD90+
XG JHH-6 CD90-
Samples

Reference List of File S1

S1. Schmittgen TD, Zakrajsek BA (2000) Effect of experimental treatment on housekeeping gene expression: validation by real-time, quantitative RT-PCR. J Biochem Biophys Methods 46: 69-81.

S2. Lin CW, Liao MY, Lin WW, Wang YP, Lu TY, et al. (2012) Epithelial Cell Adhesion Molecule Regulates Tumor Initiation and Tumorigenesis via Activating Reprogramming Factors and Epithelial-Mesenchymal Transition Gene Expression in Colon Cancer. J Biol Chem 287: 39449-39459.

S3. Kroepil F, Fluegen G, Totikov Z, Baldus SE, Vay C, et al. (2012) Down-regulation of CDH1 is associated with expression of SNAI1 in colorectal adenomas. PLoS One 7: e46665
